# The Topical Use of Earths

**Published:** 1886-05

**Authors:** 


### THE TOPICAL USES OF EARTHS.

Dr. Hewson, of Philadelphia, read before the Medical Society of the State of Pennsylvania, at its last meeting, an interesting paper on this subject. For sixteen years he has used different kinds of earths as topical applications in various diseases which involve the skin—small-pox, measels, scarlet-fever, erysipelas, etc. He has used it in form of paste, aud dusted dry upon the surface.

Clay has proven to be the best application, either dry or in paste. The reports of cases made, show the undoubted efficacy of the application on the local and constitutional conditions. To quote from his article:

"In all cases of these four diseases which have fallen under my care since 1872, I have without discrimination, always used applications of clay, and following these applications I have always noticed:

"First.—A direct and rapid reduction of temperature.

"Secondly.— An Allaying or, more positively, a dissipation of pain or distressing local sensations which belong to each of these diseases.

"Thirdly.—A diminution of the duration which is characteristic of each.

"Fourthly.—The allaying of the intensity of the general or constitutional symptoms belonging to each.

"Fifthly.—The prevention of the complications which occur so frequently as to have long been recognized as characteristic of each special disease.

"Sixthly.—The destruction of all contagiousness and power of propagation of each.

"It is but right that I should here state my mode of application, which will explain my association of measels with scarlet fever, and small-pox with erysipelas. Thus in the various forms of scarlatina and rubeola, as well as German and French measels, I have always persued the plan of dusting the powdered earth all over the cutaneous surface. There has always followed a rapid reduction of temperature, as shown by the thermometer in the patient's mouth, as well as in the axilla or groin. This reduction has generally been about 5° Fahrenheit when those diseases were in the stages of invasion, and 10°, or even as much as 15°, when the applications of the earth were not made until those diseases had progressed beyond that stage.

"In erysipelas and small-pox, I have always applied the clay directly to the skin in the form of a smooth paste, made by mixing it freely with water in a glass or china mug by means of a wooden spatula, for the reason that I wished it to hold fast and constantly to the part, a result which is always commensurate to the full whipping given to the paste. The water is here so rapidly devoured by the earth that there is always at first a marked increase of temperature of the dressing. This warmth, which is appreciable by the patient as well as by the attendant who has made the application, lasts over an hour; indeed, until the clay has become dry.

"Allowing for the difference due to the mode of application, my results show a most positive and continuous reduction of temperature following the application.

"The preliminary temperature—that taken before the first dressing was applied—has always been recognized by me as indicative of the severity of the disease according to its stage; and I have always been satisfied with that taken at the end of the first twenty-four hours as showing the effects of the earth on the disease—the greater reduction showing the greater destruction of the morbid process, or of its influence on the patient's condition.

"This reduction, during the first period of twenty-four hours, has always been greatest in measels, next in erysipelas, scarlet fever and last, in small-pox—that of measels averaging 10°, of erysipelas 8°, scarlet fever 6°, and of small-pox 5°, a difference occuring in all of them according to their being, when this application is made, in their initiative, full, or fading stages. The change of temperature on the second period of twenty-four hours has never been found to be much, if any, save that in scarlet fever it was always as great as during the first period.

"In the third period, erysipelas has always ranked first, then measles. Where the cases had been treated in this manner *ab initio*, they often showed no increase over healthy temperature, the difference being often evidently due to that of the mode of application used; erysipelas and small-pox showing more than measles and scarlet fever, because, with the former, whatever renewal of dressing had to be made, was without taking the original off, but by adding another layer of the paste on."

**Galvano Plastic Embalming.**—M. Kergovatz, of Brest, is the inventor. The body is coated with plumbago and placed in a bath of sulphate of copper connected with a battery. The body becomes thus encased in a skin or plate of copper which entirely prevents decomposition. Over this may be applied a second plating of gold or silver.

